# Histone chaperones and the Rrm3p helicase regulate flocculation in *S. cerevisiae*

**DOI:** 10.1186/s13072-019-0303-8

**Published:** 2019-09-23

**Authors:** Hollie Rowlands, Kholoud Shaban, Barret Foster, Yannic Proteau, Krassimir Yankulov

**Affiliations:** Department of Molecular and Cellular Biology, University of Guelph, Guelph, Canada

**Keywords:** Gene silencing, Gene repression, *FLO* genes, Flocculation, Histone chaperones, *RRM3*, Laboratory evolution

## Abstract

**Background:**

Biofilm formation or flocculation is a major phenotype in wild type budding yeasts but rarely seen in laboratory yeast strains. Here, we analysed flocculation phenotypes and the expression of *FLO* genes in laboratory strains with various genetic backgrounds.

**Results:**

We show that mutations in histone chaperones, the helicase *RRM3* and the Histone Deacetylase *HDA1* de-repress the *FLO* genes and partially reconstitute flocculation. We demonstrate that the loss of repression correlates to elevated expression of several *FLO* genes, to increased acetylation of histones at the promoter of *FLO1* and to variegated expression of *FLO11*. We show that these effects are related to the activity of CAF-1 at the replication forks. We also demonstrate that nitrogen starvation or inhibition of histone deacetylases do not produce flocculation in *W303* and *BY4742* strains but do so in strains compromised for chromatin maintenance. Finally, we correlate the de-repression of *FLO* genes to the loss of silencing at the subtelomeric and mating type gene loci.

**Conclusions:**

We conclude that the deregulation of chromatin maintenance and transmission is sufficient to reconstitute flocculation in laboratory yeast strains. Consequently, we propose that a gain in epigenetic silencing is a major contributing factor for the loss of flocculation phenotypes in these strains. We suggest that flocculation in yeasts provides an excellent model for addressing the challenging issue of how epigenetic mechanisms contribute to evolution.

## Background

The non-sexual aggregation of single cell organisms into clusters is referred to as flocculation or biofilm [1, 2]. In industrial yeast strains flocculation is a highly desired phenotype and in many cases can be readily activated by starvation, exposure to ethanol and/or other stressors [1, 2].

The key regulators of flocculation in *S. cerevisiae* are the *FLO* genes. They are positioned 20–40 kb away from the telomeres and encode lectin-like cell surface proteins [3, 4]. The genes contain multiple internal repeats and share significant homology with *FLO* genes in other yeast species [3, 5]. *FLO1* acts as a regulator of biofilm formation [6] while *FLO11* is known to control the switch between planktonic and filamentous growth [4]. Other members of the family include *FLO5* (paralogous to *FLO1*), *FLO9* and *FLO10* [3]. In lab strains the *FLO* genes are repressed by the Tup1/Cyc8 complex via long-range chromatin remodelling [7]. *FLO11* is reversibly switching between active and silent states, a feature reminiscent of subtelomeric genes [4, 8].

*FLO* expression and flocculation is regulated by a wide variety of mechanisms including the MAPK, TORC, SNF1 and RIM101 signalling cascades [9, 10]. Chromatin structure plays a major role in the regulation of flocculation, but details are often missing. For example, screens in the *∑1278b* strain (unlike *S288C*, *∑1278b* displays various dimorphic transitions) have shown that flocculation and filamentous growth are suppressed by mutations in components of the histone deacetylase Rpd3, the acetyl-transferase SAGA or the Ino80/Swr1p chromatin remodeler [9, 10]. In industrial yeasts, the Set1/COMPASS histone methyl transferase and the *RPD3*, *HDA1* and *HST1* deacetylases have been implicated in the repression of *FLO* genes [4, 7, 11]. Finally, a mutation in Histone H4 (H4S47C) leads to the depression of *FLO1* and flocculation [12]. Importantly, the major regulators of gene silencing in *S. cerevisiae*, the *SIR* genes, have not been pulled out in any of these screens. Instead, it has been shown that in laboratory strains the *FLO* genes are repressed by the *HST1* and *HST2* paralogs of the *SIR2* histone deacetylase [4]. Even more, in wine yeasts *SIR2* is required for the expression of *FLO11* while the acetyl transferase *SAS2* represses the transcription of *FLO5* [13].

Several histone chaperones are involved in the epigenetic transmission and maintenance chromatin structure in *S. cerevisiae* [14, 15]. The histone chaperones ASF1 and FACT are involved in both the disassembly and reassembly of nucleosomes during DNA replication. CAF-I is believed to play a central role in the re-assembly of H3/H4 tetramers behind the forks while ASF1 and Rtt106 participate in the delivery of new H3 and H4 histones [14]. ASF1 and FACT also have roles in transcription that is independent of their function in DNA replication [14]. On the other hand, the HIR and NAP1 chaperones operate in a replication-independent manner, but their precise role is not clear [14]. No reports have linked the repression of *FLO* genes to histone chaperones. However, there is solid evidence for replication-coupled chromatin assembly factors contributing to gene silencing at the sub-telomeres and the mating type *HMR/HML* loci [14, 16–19]. In addition, we have shown that Rrm3p, a DNA helicase that removes tightly bound proteins ahead of the replication forks, has a role in the mechanism of epigenetic conversions at the sub-telomere [16].

Interestingly, many laboratory *S. cerevisiae* strains contain functional copies of the *FLO* genes but do not normally flocculate, most likely because of the extensive passive selection against flocculation in favor of planktonic growth [1, 20]. We have recently noticed that mutations in various histone chaperones promote flocculation-like phenotypes. In this manuscript, we report our extensive analyses of these observations.

## Results

### Flocculation-like phenotypes in laboratory strains

While analysing epistatic interactions of histone chaperones with the *RRM3* helicase, we noticed that some mutant strains produced clusters in liquid cultures. This was surprising as all mutations were in haploid *BY4742* or *W303* backgrounds, which do not flocculate under normal laboratory conditions. We systematically compared these and other phenotypes of all strains listed Table 1 plus about another 120 strains with mutations in various genes (not shown).

**Fig. 1:**
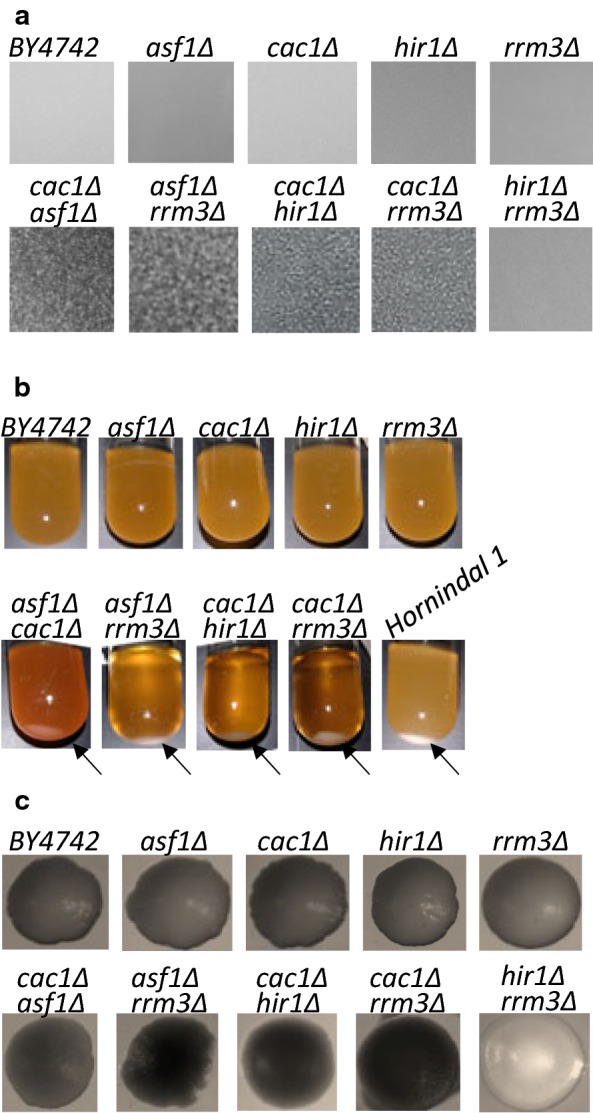
Flocculation-like phenotypes in laboratory strains. **a** Liquid cultures (strains shown on top) were poured in Petri dishes and pictures taken with a digital camera without magnification. **b** Liquid cultures were grown on a spinning wheel and rested for 10 min before pictures were taken. **c** Pictures of colonies were taken by an inverted microscope at ×2.5 magnification

No flocculation-like phenotypes were observed in any of the single deletion mutants in *BY4742* and *W303* genetic background, thus reiterating the notion that these laboratory strains have lost the ability to form biofilm (Fig. 1a). On the other hand, when liquid cultures were grown on a spinning wheel visible clusters of cells were observed in strains with some, but not all combinations of double deletions of *cac1*, *asf1*, *hir1* and *rrm3*. As already mentioned, *CAC1*, *ASF1* and *HIR1* encode for histone chaperones engaged in the assembly of nucleosomes [14] while *RRM3* encodes a helicase that relieves replication pausing [21]. Flocculation was apparent as visible clusters in liquid cultures as compared to the uniform turbid appearance of the non-flocculating strains (Fig. 1a). Even more, the cultures formed pellets shortly after removal from the wheel (Fig. 1b). The levels of sedimentation of these laboratory strains were comparable to the sedimentation of a *wild type* beer strain with a well documented flocculation phenotype (*Hornindal 1* [22]) (Fig. 1b). In all cases, these clusters were dispersed by exposure to 10 mM EDTA (not shown) as previously observed in many wild type strains [23].

It has been reported that in feral and industrial strains flocculation is often accompanied by altered colony morphology, stronger adhesion to agar and cell cycle arrest [6]. However, we noticed that our flocculating strains formed even lustre colonies similar to the ones formed by non-flocculating strains (Fig. 1c) with no evidence of stronger adhesion to agar as compared to the isogenic wild type strains (not shown). We also found no correlation between growth rates of the strains, their progression through the cell cycle (Additional file 1) and flocculation (Fig. 1a). Even more, individual isolates of some strains displayed various levels of flocculation as judged by the observation of clusters under a microscope (not shown) while growth rates were the same.

We concluded that double deletion mutants in the *BY4742* and *W303* laboratory strains promote apparent cell aggregation, but not a full display of other flocculation-related phenotypes observed in some wild type *S. cerevisiae* strains.

### Recombination of *FLO* genes, spontaneous mutations and sensitivity to DNA damage do not correlate to flocculation-like phenotypes

Prior studies have reported variations in the number of intragenic repeats of *FLO* genes of industrial yeasts and have suggested that homologous recombination and length variations could have phenotypic and evolutionary implications [5, 24]. In addition, it has been previously reported that *cac1∆* and *rrm3∆* strains have elevated spontaneous mutation rates and sensitivity to DNA damage [16, 17, 25]. For this reason, we analysed the length of *FLO* genes in our laboratory strains as in [5]. We also looked at the frequency of spontaneous mutations as measured by the canavanine resistance fluctuation assay [25] and at the sensitivity to DNA damage as measured by exposure to Methyl Methane Sulfonate (MMS). We observed length variation only in *FLO10* in the *cac1∆asf1∆* strain, but not in any of the *FLO* genes in any of the flocculating laboratory strains (Additional file 1: Figure S3). The canavanine resistance assay measures the rate of spontaneous loss-of-function mutations in the *CAN1* arginine transporter gene and was performed only in the strains that do not already harbor the *can1*-*100* mutation. These limited in scope assays indicated a modest increase in spontaneous mutation rates in *rrm3∆tof1∆* and *cac1∆tof1∆* which do not flocculate. In all other tested strains the mutation rates were indistinguishable from the wild type *BY4742* strain (Additional file 1). Finally, there was no correlation between sensitivity to MMS (Additional file 1) and flocculation of the strains.

We concluded that the flocculation in our laboratory strains is not related to any of these earlier reported phenotypes and characteristics in various single deletion mutants.

### Elevated expression of *FLO* genes in flocculating strains

**Fig. 2:**
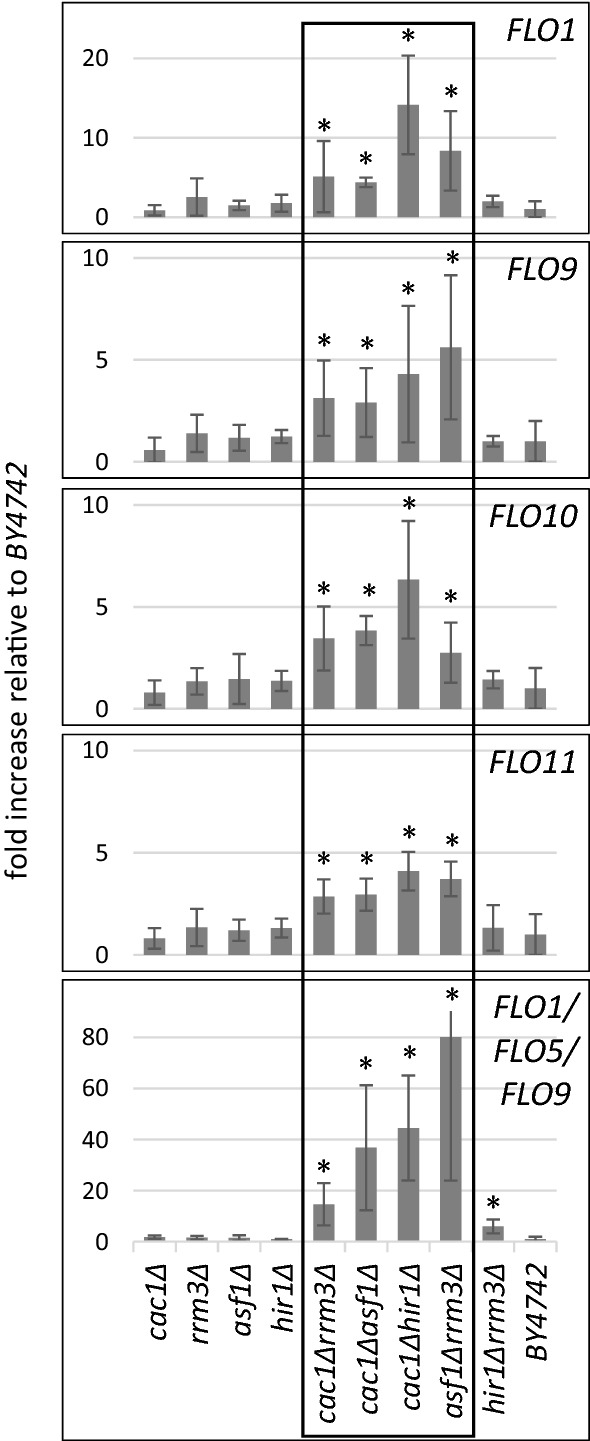
Elevated *FLO* gene expression and length variation in *FLO* genes. Total RNA was isolated from the strains shown on the horizontal axis. The abundance of *FLO* RNAs was measured by qRT-PCR and normalized to *ACT1.* The signals in the *BY4742* strain were given a value of “1”, the signals from the other strains were normalized to the signals from *BY4742* and plotted. The bottom panel represents analyses with primers that amplify *FLO1*, *FLO5* and *FLO9*. Each bar represents the average value three to five independent biological replicas with each strain/primer pair. Asterisks represent difference between the average signals from mutant strains as compared to *BY4742* at *p* < 0.05

In industrial yeasts, the *FLO* genes are repressed in planktonic cultures and active in flocculating ones [4, 6]. We asked if the flocculation phenotype in our strains could be attributed to the elevated expression of the *FLO* genes. RNA was isolated from four flocculating and four non-flocculating strains and analysed by qRT-PCR with primers specific for each of *FLO1*, *FLO9*, *FLO10* and *FLO11*. Because of the highly repetitive nature of the high degree of homology between the *FLO* genes, the primers for *FLO5* also amplify the RNAs produced by *FLO1* and *FLO9*. Three to five independent experiments were performed with each strain/primer combination and the measured amounts in the mutants were compared to the expression of the *FLO* genes in the isogenic *BY4742* strain. The analyses showed 1- to 2-fold increase in the expression of *FLO1* and *FLO9* and 2- to 3-fold increase in *FLO10* and *FLO11*, respectively, in the non-flocculating single deletion mutants *cac1∆*, *asf1∆*, *rrm3∆* and *hir1∆* (Fig. 2). On the other hand, the expression of all *FLO* genes was 4- to 12-fold higher in the flocculating double deletion mutants. The *FLO1/FLO5/FLO9* primers detected 10- to 80-fold increase in the abundance of these RNAs (Fig. 2). While the overexpression of *FLO* genes in flocculating strains was consistently observed, the magnitude of effects differed between individual experiments (Fig. 2). While we cannot confidently explain these fluctuations, we suspect that *FLO* gene expression varies in individual cultures and that this variation could be an important component in the adaptation/flocculation strategy of the cells. This notion is consistent with the observed difference of flocculation between individual isolates of the same strains, as mentioned above. Regardless of the nature of these variations, we observed a consistent correlation between the flocculation phenotype and the higher expression of the *FLO* genes.

### Hyper-acetylation of histones H3 and H4 at the *FLO* loci in the flocculating strains

**Fig. 3:**
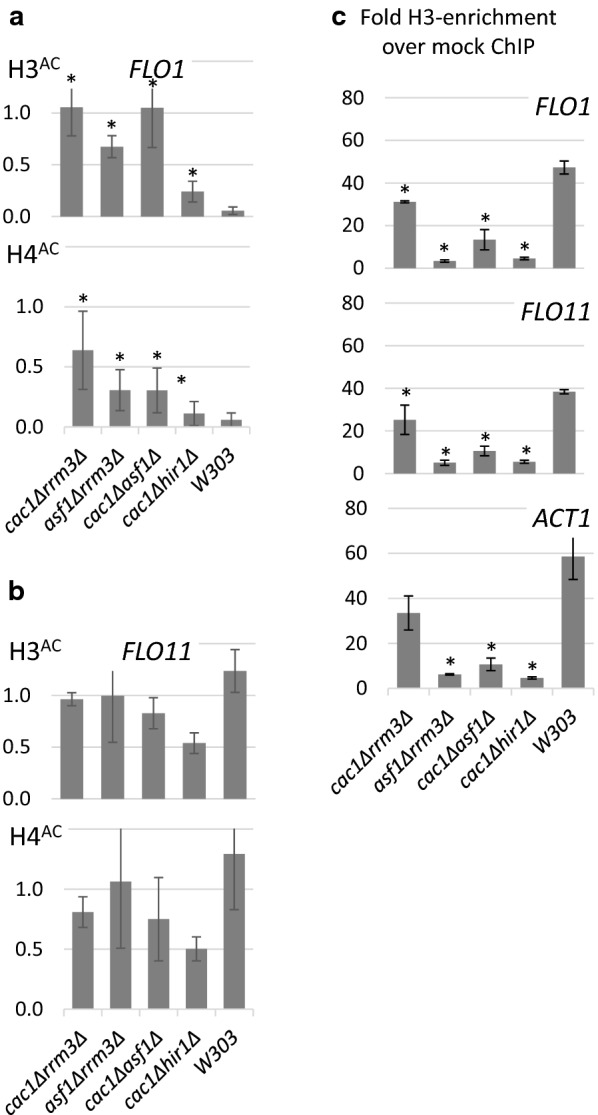
Histone H3 abundance and H3/H4 hyperacetylation at *FLO* gene loci. Chromatin extracts from the strains shown below the horizontal axis were immunoprecipitated with antibodies against Histone H3, acetylated Histone H3 (H3^AC^) and acetylated Histone H4 (H4^AC^). The precipitated DNA was amplified with primers for the promoter regions of *ACT1*, *FLO1* and *FLO11*. The signals were normalized to *ACT1* and then to the anti-H3 immunoprecipitates and plotted. The bars represent the average of two to three independent experiments with each strain/primer pair. Asterisks represent difference between the average signals from mutant strains as compared to *BY4742* at *p* < 0.05. **a** H3/H4 acetylation at the *FLO1* promoter. **b** H3/H4 acetylation at the *FLO11* promoter. **c** H3 abundance at ACT1 and the *FLO1* and *FLO11* promoters

Earlier studies have shown that the repression of *FLO1* is dependent on the *HST1*, *HST2*, *HDA1* and *RPD3* histone deacetylases and that *FLO1* de-repression is associated with the hyperacetylation of Histones H3 and H4 at its promoter [7, 26]. While the mutants we have analysed so far do not encode histone deacetylases, we reasoned that their effects could nevertheless be mediated by hyperacetylation of Histones H3 and H4 at the *FLO* genes promoters. We addressed this question by Chromatin-Immuno-Precipitation (ChIP) with anti-H3, anti-H3^AC^ and anti-H4^AC^ antibodies followed by quantitative PCR with primers for the promoter regions of *FLO1* and *FLO11*. Consistent with earlier observations, at the *FLO1* promoter the flocculating strains produced 2–11 times higher signal with the anti-H3^AC^ antibody and 3–6 times higher signal anti-H4^AC^ antibodies, as compared to the *BY4742* strain (Fig. 3a). On the other hand, there was no difference in the acetylation of histones at the *FLO11* promoter between the flocculating strains and *BY4742* (Fig. 3b). We suspect that we were not able to detect difference in H3/H4 acetylation at the *FLO11* promoter because *FLO11* switches between active and silent states [4] thus producing a higher basal signal in ChIP experiments. It has also been shown that *FLO1* remains active while *FLO11* is repressed depending on the abundance of Tup1p and Cyc8p [27]. Additionally, it remains possible that our RT-qPCR (see above) and GFP expression driven by the *FLO11* promoter (see below) analyses are sensitive enough to reveal transient increases in *FLO11* expression but not temporary acetylation changes by ChIP assays.

We also quantified the H3-ChIP signals at *ACT1* and the *FLO1*, *FLO11* promoters. The results showed that in the *cac1∆asf1∆*, *asf1∆rrm3∆* and *cac1∆hir1∆* strains the H3-ChIP signals decline relative to *W303* at these three loci with a similar but less pronounced effect in *cac1∆rrm3∆* (Fig. 3c). We also tested the sensitivity of the chromatin in these strains to micrococcal nuclease (MNase) (Additional file 1: Figure S7). In agreement with the H3-ChIP data, we observed substantially higher sensitivity to MNase in *cac1∆asf1∆*, *asf1∆rrm3∆* and *cac1∆hir1∆* cells and modest increase in *cac1∆rrm3∆* cells relative to *W303*. These results are consistent with earlier observations showing increased sensitivity to nucleases in *cac1∆* and decreased sensitivity in *asf1∆* [28]. However, the deletion of additional genes in our strains seems to exacerbate the altered sensitivity to nucleases and lead to a profound de-repression of the *FLO* genes.

Taken together, the ChIP, MNase sensitivity and RT-PCR data point to a lower nucleosome density and higher H3/H4 acetylation at the *FLO* promoters that contributes to the loss of repression of the *FLO* genes.

### Epistatic interactions of *CAC1*, *ASF1* and *HIR1* with *HDA1*

**Fig. 4:**
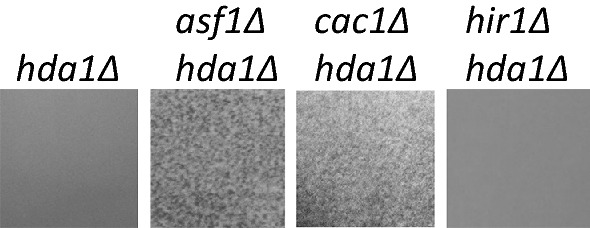
Epistatic interactions between *CAC1*, *ASF1*, *HIR1* and *HDA1.* Liquid cultures (strains shown on top) were poured in Petri dishes and pictures taken a digital camera without magnification

We reasoned that if histone chaperones are indeed playing a central role in the transmission and maintenance of repressive chromatin at the *FLO* loci, then *CAC1*, *ASF1* and *HIR1* would genetically interact the histone deacetylases *HDA1* or *RPD3* to promote flocculation phenotypes. We constructed haploid *cac1∆hda1∆*, *asf1∆hda1∆* and *hir1∆hda1∆* strains, but were not successful in producing double deletion mutants with *RPD3* with any of these genes. We observed apparent flocculation in the *cac1∆hda1∆* and *asf1∆hda1∆* strains, but not in the *hir1∆hda1∆* strain (Fig. 4). Similarly, no flocculation was observed in the *hir1∆rrm3∆* strain. These observations suggest that *HIR1* is not normally involved at the replication forks, but might have a role in *FLO* gene repression by yet unknown mechanism.

### Flocculation in single deletion mutants is induced by nitrogen starvation or by inhibition of histone deacetylases

**Fig. 5:**
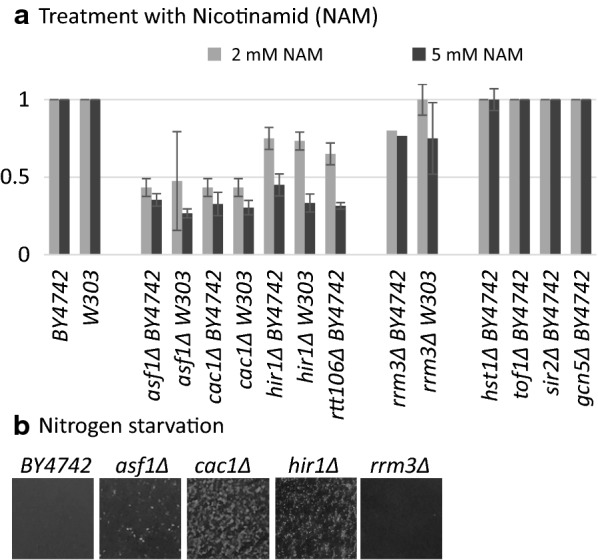
Nitrogen starvation and nicotinamide (NAM) induce flocculation. **a** Cells were grown in low nitrogen liquid medium for 2 days, then poured in Petri dishes and pictures taken with a digital camera without magnification. **b** The strains shown under the horizontal axis were grown overnight in the presence of 0 (control), 2 and 5 mM Nicotinamide (NAM). Sedimentation rates were calculates as the time needed for the clearing of the upper 50% of the culture (T^S50^) in the NAM treated samples divided by T^S50^ in the corresponding NAM-free cultures. The bars represent the average of 2–3 measurements with each strain. The lack of errors in the *BY4742* and *W303* bars represent no lack of detectable difference in the presence of NAM

Nicotinamide (NAM) is a potent competitive inhibitor of NADH^+^-dependent histone-deacetylases [29]. Cells exposed to NAM display reduced silencing of subtelomeric genes and at the mating type loci [30], however, its effect on flocculation has not been reported. We tested this possibility by growing liquid cultures of various strains in the presence of 0, 2 and 5 mM NAM. The cultures were then rested for 30 min and the rate of sedimentation in the NAM-treated relative to the non-treated samples was measured. Three cultures per strain were scored on 3 different days. Flocculation was further confirmed by light microscopy as in Fig. 1. Sedimentation scores are shown in Additional file 1: Table S2 and the results are summarized in Fig. 5a. In the presence of 5 mM NAM we observed 3–4 times faster sedimentation rates in *cac1∆* and *asf1∆* strains and 2 times faster sedimentation rates in *hir1∆* and *rtt106∆* strains in both *W303* and *BY4742* background (Fig. 5a). The *rrm3∆* strain showed less than twofold faster rates. All other strains tested, including *BY4742*, *W303*, *hst1∆*, *tof1∆*, *sir2∆*, *gcn5∆* (Fig. 5a) or the already flocculating double deletion mutants (not shown) revealed no apparent detectable increase in sedimentation rates in the presence of NAM.

So far we have shown that strains with single deletions do not produce floccules but do so in combination with the deletion of other genes or upon incubation with NAM (Figs. 1, 5a). On the other hand, it has been reported that stress and starvation induce flocculation in wild type yeast strains [1]. We asked if the single deletion mutants would produce floccules in media containing low nitrogen or low carbon source. The low carbon medium had little effect on the flocculation of any of these strains (not shown). On the other hand, upon nitrogen starvation we readily observed flocculation in the *cac1∆*, *asf1∆* and *hir1∆*, but not in the isogenic *BY4742* strain or in the *rrm3∆* strain (Fig. 5b).

These experiments indicated that the repression of the *FLO* genes is already compromised in *cac1∆*, *asf1∆*, *hir1∆* and *rtt106∆* mutants and can be further revealed by nitrogen starvation or treatment with NAM.

### Loss of the association of CAF-1 with PCNA induces flocculation

**Fig. 6:**
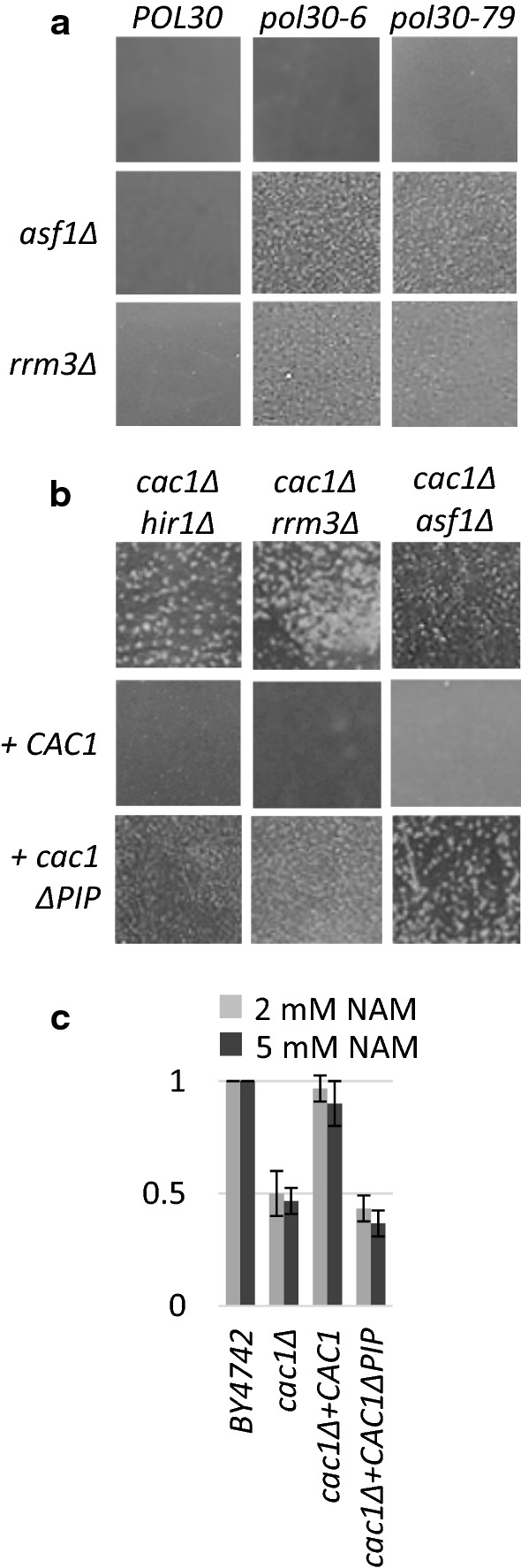
Loss of association of CAF-1 with PCNA promotes flocculation. **a** *ASF1* and *RRM3* were deleted in *POL30*, *pol30*-*6* and *pol30*-*79* strains. The cells were grown in liquid cultures, poured in Petri dishes and pictures taken with a digital camera without magnification. **b** *cac1∆hir1∆*, *cac1∆rrm3∆* and *cac1∆asf1∆* strains were transformed with plasmids expressing Cac1p or Cac1p with a destroyed PCNA-Interacting Peptide (*cac1∆PIP*). The cells were grown in liquid cultures, poured in Petri dishes and pictures taken with a digital camera without magnification. **c** A *cac1* strain was transformed with plasmids expressing Cac1p or Cac1p with a destroyed PCNA-Interacting Peptide (*cac1∆PIP*) and the cells were grown in liquid cultures containing 2 and 5 mM nicotinamide (NAM). Sedimentation rates were measured and plotted as in Fig. 5

We asked if our observations depend on the activity of CAF-1 in a replication-dependent or independent fashion. We used two strains that harbor mutations in the replication clamp PCNA (*pol30*-*6* and *pol30*-*79*), which are known to significantly reduce its association with CAF-1 [31]. We reasoned that these PCNA mutations could produce effects similar to these seen in *cac1∆* mutants. As expected, the deletion of *RRM3* and *ASF1* in the *pol30*-*6* and *pol30*-*79*, but not *POL30* strains produced apparent flocculation (Fig. 6a). Interestingly, the deletion of *RRM3* produced a stronger effect in the *pol30*-*6* mutant and the deletion of *ASF1* produced a stronger effect in the *pol30*-*79* mutant (Fig. 6a). At present, we cannot explain the differences between the two *pol30* alleles. Next, we complemented the *cac1∆hir1∆*, *cac1∆rrm3∆* and *cac1∆asf1∆* strains with *CAC1* or *cac1∆PIP* with a destroyed PCNA Interacting Peptide (PIP), which is known to preclude the association of CAF-1 with PCNA and CAF-1 mediated replication-coupled chromatin assembly in vitro [32]. In Fig. 6b we show that complementation by *CAC1* substantially reversed their flocculation phenotype while *cac1∆PIP* had no effect. Finally, we complemented a *cac1∆* strain with *CAC1* and *cac1∆PIP* and exposed them to Nicotinamide (NAM). In Fig. 6c we show that NAM-induced rates of sedimentation, similar to what is observed in *cac1∆1* mutants in the strain complemented with *cac1∆PIP* but not by *CAC1*. Together, these experiments demonstrated that the inability of CAF-1 to associate with PCNA can induce flocculation.

### Variegation and loss of *FLO11* silencing in a flocculating strain

**Fig. 7:**
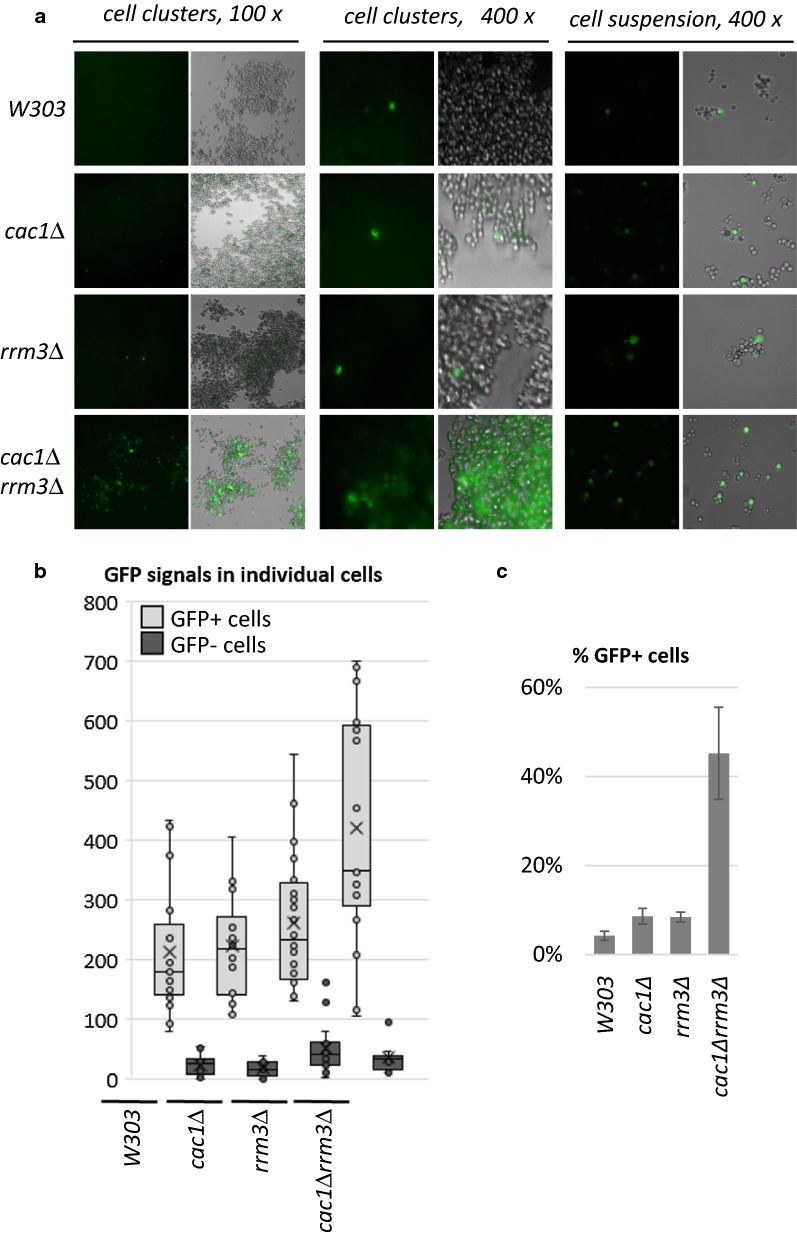
Variegated expression of *FLO11*-*GFP*. **a** Cell clusters (left hand and middle panels) were produced by serial dilution of liquid cultures in 96-well trays and incubation for 24 h with no shaking. Cell suspensions were also dispersed and spread on slides (right hand pael). Images were taken with a Leica DM 6000B microscope and processed with Velocity™ software. **b** GFP signals in at least 50 individual cells were acquired and plotted. **c** Cells that display at least 3 times higher GFP signals relative to the signal in GFP− cells in **b** were calculated and plotted

It has been previously demonstrated that a wild type yeast strain expresses *FLO11* in a variegated fashion producing patches of GFP+ and GFP− cells when the gene is tagged with GFP [4] and that *FLO11* is contributing to filamentous growth [33]. We, therefore, asked if flocculation can be correlated to the variegated expression of *FLO11*. We replaced the *FLO11* ORF with GFP as in [33] and tested the expression pattern of GFP in a flocculating (*cac1∆rrm3∆*) and two non-flocculating laboratory (*cac1∆* or *rrm3∆*) strains in *W303* genetic background. Briefly, cells were dispersed, serially diluted in 96-well trays and incubated without shaking for 24 h. Sparsely populated wells with isolated cell clusters were then analysed by fluorescent microscopy. In *cac1∆*, *rrm3∆* and the control *W303* strains we observed a very low number of isolated GFP+ cells (Fig. 7a). In contrast, the flocculating *cac1∆rrm3∆* displayed clusters of cells with various levels of green fluorescence as well as cells with no apparent fluorescence (Fig. 7a).

Because cell clusters do not allow for a precise focusing and measurement of the GFP signals, the strains were grown in suspension for 24 h, dispersed by vigorous vortexing/pipetting and spread on microscope slides. GFP signals were acquired for both individual GFP+ and GFP− cells and used for the measurement of GFP+ signals in individual cells and for the calculation of the percent of GFP+ cells in each strain. In agreement with the observations in cell clusters (Fig. 7a), these measurements showed that the signals from individual *cac1∆rrm3∆* cells significantly vary, but on average were substantially higher than the signals in *cac1∆*, *rrm3∆* or the control *W303* strains (Fig. 7b). We suspect that the difference in the detected levels of *FLO11* RNA (Fig. 2) and GFP (Fig. 7) could be caused by the stability of GFP. Next, the percent of GFP+ cells was calculated. GFP+ cells were defined as cells that have 3 times higher green fluorescence as compared to the average fluorescence from the GFP− cells. Based on these criteria, we show about 45% GFP+ cells in the *cac1∆rrm3∆* strain as compared to 8%, 9% and 4% in the *cac1∆*, *rrm3∆* and *W303* strains, respectively.

We concluded that flocculation in the *cac1∆rrm3∆* strain is accompanied by a gain of *FLO11* gene expression in about half of the cells in the culture. We also concluded that the expression of GFP as driven by the *FLO11* promoter varies between individual cells. Finally, because we did not observe large patches of GFP+ and GFP− cells in the cell clusters (Fig. 7a, left panel), we suspect that the conversion rates between active and silent *FLO11* are high and cannot be assessed by the methods we have used in the past for the analysis of conversion rates at the telomeres [25].

### Correlation of repression of *FLO* genes and the sub-telomeric and the mating type loci in laboratory yeast strains

Next, we looked at data from earlier publications by us and others [4, 16, 19, 25, 34] to compare flocculation phenotypes and *FLO* gene silencing to the level of gene silencing at the telomeres and the mating type loci (Table 1). Consistent with earlier observations [4, 34] and this study, *SIR3* was required only for gene silencing at the sub-telomeres and the mating type loci and had no effect on the expression of the *FLO* genes. At the same time, the single and double deletions of histone chaperones and *RRM3* seemed to have a similar magnitude of effect on all these loci. For example, single deletions of *CAC1*, *ASF1*, *HIR1* and *RRM3* do not or only transiently de-repress the mating type loci [16, 19, 25, 34], moderately reduce the repression of *FLO* genes (this study) and moderately reduce gene silencing at the telomeres [16, 25]. In comparison, double deletions of these genes cause a measurable loss of silencing at the mating type loci ([19, 25, 34] and Additional file 1: Figure S6), significant de-repression of the *FLO* genes (this study) and severe loss of silencing at the telomeres [16, 25].

**Table 1:** Comparison of the levels of repression at the *FLO* loci, *VIIL* telomere and the mating type loci

Strain	Flocculation	Increase in *FLO* gene expression	Silencing at the *VIIL* telomere (%FOA^R^ cells)	Silencing at the *HML/HMR* mating type loci
*BY4742*	No	n/a	66% (25)	No loss [34]
*sir3∆*	No	< 1*x*	< 0.1%	Loss [34]
*cac1∆*	No	1–2*x*	5% (16)	No loss [25, 34], transient loss [19]
*rrm3∆*	No	1–2*x*	10% (16)	No loss (Additional File 1)
*asf1∆*	No	1–2*x*	9% (25)	No loss [34] transient loss [19]
*hir1∆*	No	1–2*x*	53% (25)	No loss [25]
*cac1∆asf1∆*	Yes	> 5*x*	< 1% (25)	Loss [25, 34]
*cac1∆hir1∆*	Yes	> 5*x*	< 1% (25)	Loss [25]
*cac1∆rrm3∆*	Yes	> 5*x*	< 0.1% (16)	Loss (Additional File 1)
*asf1∆rrm3∆*	Yes	> 5*x*	< 0.1% (16)	Loss (Additional File 1)
*hir1∆rrm3∆*	No	> 5*x*	11% (16)	No loss (Additional File 1)
*orc5*-*1*	Yes	> 5*x*	< 1% (25)	Loss [47]
*sas2∆*	No	n/a	4% (25)	No loss (Additional File 1)
*tof1∆*	No	n/a	26% (16)	n/a
*mcm5*-*461*	No	n/a	4% (25)	n/a
*bob1*-*1cdc7∆*	No	n/a	3% (25)	n/a
*cac1∆tof1∆*	No	n/a	n/a	n/a
*cac1∆sas2∆*	No	n/a	n/a	n/a
*tof1∆rrm3∆*	No	n/a	n/a	n/a
*hda1∆*	No	n/a	n/a	n/a
*hst1∆*	No	n/a	n/a	n/a
*rpd3∆*	No	n/a	n/a	n/a

Data from this study and [4, 16, 19, 25, 34] were used to compare the magnitude of de-repression in different mutants. The de-repression of *FLO* genes is based on the data in Fig. 2 and expressed as the average fold increase in the expression of *FLO1*, *FLO5*, *FLO9*, *FLO10*, *FLO11* in the mutants relative to *BY4742* in both exponentially growing and saturated cultures. The sub-telomeric gene repression is measured by the routine Telomere Position Effect (TPE) assay [46] and is displayed as per cent of 5-Floro-Orotic-Resistant (FOA^R^) cells, which carry *URA3* adjacent to the *VIIL* telomere. The repression of mating type loci is measured by the expression of a *GFP* reporter inserted in *HMR* [25, 34]; by the CRASH assay, which measure transient expression from the *HML* locus; or by measurement of the mating efficiency (this study) “*No loss*” means less than 0.01% GFP+ cells and “*loss*” means more than 10% GFP+ cells. The “*transient*” and “*strong*” are used exactly as in [19]

*n/a* not available in any of the listed sources

### Flocculation phenotype in an ancestral strain

**Fig. 8:**
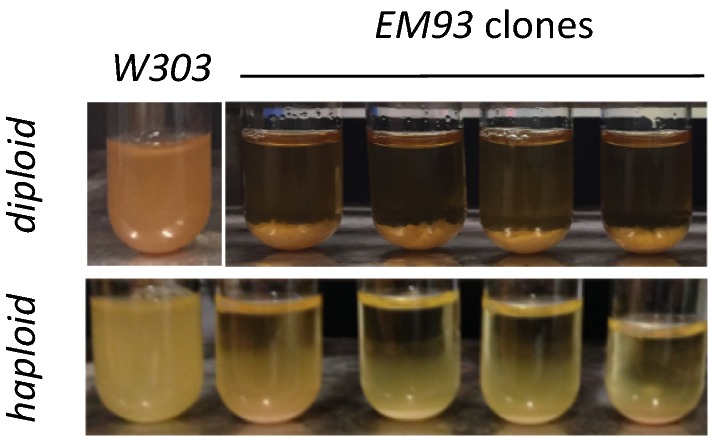
Flocculation phenotype in an ancestral strain. Four individual clones from isolated spores of the ancestral strain *EM93* or from *EM93 ho∆* strain produced in the lab were grown for 60 generations, the tubes were then rested for 5 min and pictures were taken

Next, we asked if an ancestral strain (*EM93*, [35]) used to produce *S288C* and eventually *BY4742* and *W303* is flocculating under normal laboratory conditions. We obtained an old stock of *EM93*, which was heavily flocculent. We produced four independent clones form dissected individual spores and grew them for at least 60 generations. Because the *EM93* strain is HO+ and is capable of switching its mating type, by the time of the assessment of flocculation these cultures have become diploid as confirmed by test growth in sporulation medium and observation of tetrads. All *EM93* clones displayed heavy flocculation (Fig. 8). Next, we disrupted *HO* and produced four haploid *EM93* clones, which also displayed flocculation, but not as strong as the diploid clones. These observations indicated that the original feral strain had the ability to flocculate in both haploid and diploid state, however, this phenotype was lost through the subsequent breeding and selection for planktonic growth [35].

## Discussion

Here, we report the partial reconstitution of flocculation in laboratory yeast strains, which have lost this phenotype by continuous passive selection for planktonic growth [1]. The precise reasons for the loss of flocculation are not clear. It has been reported that in the *W303* and *BY4742* strains *FLO8* (a transcription factor for the *FLO* genes) harbors an inactivating point mutation [20], while variation in *FLO11* were correlated to the phenotype of *Σ1278b* [36]. We show that, in addition to this and possibly other gene mutations, the loss of flocculation is due to a significant gain in *FLO* gene silencing by epigenetic means. In support, we tested 120 mutant laboratory strains (not shown) and demonstrate that flocculation phenotypes can be reconstituted by the deletion of histone chaperones, histone deacetylases and a DNA helicase that relieves replication pausing (Fig. 1, Additional file 1: Table S2 and data not shown). We also show that the *EM93* strain, which is ancestral to *S288C* and *W303*, has apparent flocculation phenotype when grown under normal laboratory conditions. These experiments do not reveal exactly how the feral strain has lost its flocculation trait. In comparison, a continuous culturing and analysis of other feral strains has shown their ability to produce various colony morphologies and flocculation phenotypes [37]. It is quite possible that a similar process of adaptation/domestication has produced the non-flocculating phenotype of *W303*. Our study suggest that epigenetics could be an important factor of this and other similar adaptive processes.

While the involvement of histone chaperones in the repression of *FLO* genes is not far fetched, the role of *RRM3* calls for a special consideration. It encodes a DNA helicase dispensable for DNA replication over most of the genome, but necessary to relieve pausing at sites of tightly bound proteins [38]. Such pausing sites are frequent in the subtelomeric regions and at the mating type loci [38], but direct evidence for replication pausing at the *FLO* genes is not available (Dr. Ivessa, personal communication). It remains possible that the epistatic interactions of *RRM3* with *CAC1* and *ASF1*, but not *HIR1* (Figs. 1, 4) reflect the susceptibility of the *FLO* loci to replication pausing and subsequent chromatin perturbation in the absence of chaperones. In this line of thought, both CAF-1 and Rrm3p physically interact with the replication fork clamp PCNA through a conserved PIP (PCNA-Interacting Peptide) motif [39, 40]. Here, we have shown that the flocculation correlated to the ability of CAF-1 to associate with PCNA, thus strongly suggesting that at least the effect of CAF-1 is related to its activity at the replication forks. Our finding may indicate that Rrm3p modulates the association of CAF-1 with PCNA and exacerbates the compromised reassembly of chromatin at paused replication forks [14]. We should also mention that the deletion of *RRM3* did not enhance flocculation upon nitrogen starvation or in the presence of NAM (Fig. 5). Further studies will be required to refine the role of Rrm3p in these processes and its precise contribution to chromatin maintenance and to the gene repression.

The role of *HIR1* in the repression of *FLO* genes is also unclear. We have observed apparent flocculation phenotypes in the *cac1∆hir1∆* strain and in the *hir1∆* strain when exposed to nitrogen starvation or NAM (Figs. 1, 5). On the other hand, the *hir1∆rrm3∆* and *hir1∆hda1∆* strains showed no phenotype (Fig. 4). It remains possible that the effects of *HIR1* deletion are related to the proposed secondary role of HIR in replication-coupled nucleosome assembly in the absence of CAF-I [32]. Alternatively, *HIR1* has a limited effect on the repression of *FLO* genes that can be revealed only after compromising their repression by other means.

Most of the studies on gene repression in budding yeast have focused on *SIR*-dependent silencing at subtelomeric and mating type loci [8, 41]. At these positions gene silencing is executed by *cis*-acting silencers that serve as an assembly point of the Sir3/4 proteins, which recruit the Sir2p histone deacetylase and initiate the spreading of histone deacetylation away from the silencers [41]. As already mentioned, *FLO* genes are silenced by the binding of the Tup1/Cyc8 and Sfl1 repressors upstream of the promoters of these genes and utilize the *HDA1*, *HST1* and *RPD3* but not the *SIR2* histone deacetylase [4, 7, 42]. It can be said, within the limitations of our current knowledge, that the regulation of the *FLO* genes and the regulation of gene silencing at the telomeres and the mating loci represent different mechanisms. At the same time, multiple studies have linked *SIR*-dependent gene silencing to various DNA replication factors and histone chaperones (reviewed in [14]). Here, we compared the findings in this manuscript to previously published studies (Table 1). We found that the deletions of individual genes or combination of genes show a similar trend of loss of silencing at the mating type loci and sub-telomeres and the loss of repression of the *FLO* genes. We have also found that treatment of selected mutants with NAM increases flocculation and decreases the silencing at the mating *HMR* locus (KS, not shown). These correlation analyses support the idea that despite the difference in the mechanisms that establish repression, all these loci share similar requirement for histone chaperones and *RRM3* (Table 1).

Another similarity worth mentioning is that subtelomeric genes, partially de-repressed mating loci and at least *FLO11* are meta-stable, meaning that they infrequently switch between active and silent state [1, 8]. Here, we have shown that the elevated expression of *FLO11* in a flocculating laboratory strain reflects a wide range of the abundance of Flo11p in individual cells. We are not certain if *FLO11* alone or all *FLO* genes variegate. However, it is tempting to speculate that if all *FLO* genes variegate, they would provide a wide repertoire of cells adhesion patterns, which in turn would aid the adaptation in response to changes in the environment. It is also possible that the varying abundance of GFP as driven by the *FLO11* promoter reflects a competition between all *FLO* genes for regulatory transcription and chromatin factors and that this competition is a key to the variegated expression of these genes. This matter deserves a special attention in future studies.

## Conclusions

We show that the deregulation of chromatin transmission and maintenance is sufficient to reconstitute flocculation in laboratory yeast strains. These observations suggest that in these strains lack of flocculation is mediated by epigenetic repression at *FLO* gene loci. Our paper highlights the *FLO* genes as attractive loci for future investigation of how epigenetic silencing can drive adaptation and the acquisition of novel phenotypes. Finally, the adverse effects of yeast pathogens like *C. albicans* and *C. glabrata* are linked to dimorphic transitions, which involve genes homologous to the *FLO* genes in *S. cerevisiae* [1, 43]. Hence, our study might indirectly shed light on the epigenetic basis of this significant health problem.

## Materials and methods

### Yeast strains

The strains used in this study are listed in Additional file 1: Table S1. All assays were conducted with haploid strains in *BY4742* and *W303* background. Double deletion mutants were produced by routine mating and sporulation. Cells were routinely grown on YPD or SC dropout plates at 30 °C with the exception of temperature-sensitive mutants which were maintained at 23 °C. Liquid cultures were grown on a spinning wheel, not a shaker, to better reveal flocculation. Growth rates of all cultures were measured in *ThermoScientific Multiskan G0* instrument. For the nitrogen starvation experiments the strains were grown for 2 days at 30 °C on a spinning wheel in SC medium containing 1% YNB. For the carbon starvation assays SC medium was supplemented with 0.1% glucose and 2% glycerol.

### Assessment of flocculation phenotypes

Flocculation was determined by visual observation of cell clusters (Fig. 1) and cell aggregation was confirmed by light microscopy. Sedimentation rates were estimated by resting culture tubes and measuring the time needed for the clearance of the upper 50% of the culture (T^S50^). The measurement of sedimentation rates in the presence of nicontinamide (NAM) was conducted by dividing the T^S50^ for the cultures grown in NAM divided by T^S50^ in the corresponding NAM-free cultures.

### RT-PCR

RNA was isolated with TRIzol™ solution according to manufacturer’s directions, except that samples were vortexed for 5 min (30 s on, 30 s off) in the presence of equal volume glass beads and precipitated with Ethanol. RNA concentration and purity was determined by *ThermoScientific NanoDrop 8000.* cDNA synthesis was performed using Applied Biosystems High-capacity cDNA Reverse Transcription Kit. Quantitative PCR was carried out using *Applied Biosystems StepOne™Plus* thermocycler and PowerUp SYBR Green Master Mix. 6.25 ng of cDNA was added to each reaction and each sample was analyses in triplicates. Quantitative expression values were determined using the ΔΔCq method, wherein the average Cq for each *FLO* gene was normalised to Cq values for *ACT1*. Three to five independent experiments were performed with each strain/primer combination and average values, standard deviations and *t* tests were calculated in Microsoft Excel^®^. The ΔCq values for each strain/primer combination were normalised to ΔCq values obtained for *BY4742* cells and fold expression was calculated as 2^−ΔΔCq^ and shown in a bar graph format.

### Chromatin immunoprecipitation

50 mL cultures were grown to OD_600_ ~ 0.8, pelleted and washed once in 1 mL of LB buffer (20 mM Tris pH 7. 5 mM EDTA, 140 mM NaCl). Cells were resuspended in 300 μL LB plus 1.5× protease inhibitors (G BioSciences ProteaseArrest™ Yeast/Fungal) and lysed with 500 μL of glass beads for 18 cycles of 30 s ON/30 s OFF with a VWR Pulsing Vortex Mixer. Lysates were spun at 13,000 rpm for 15 min, the supernatant was removed and the pellet resuspended in 500 μL of MNB (200 mM CaCl_2_, 20 mM Tris pH 7.5, 140 mM NaCl, 1× protease inhibitors). 4 U of MNase was added and the samples were incubated for 5 min at 37 °C followed by the addition of 1/10 volume of STOP (25 mM EDTA, 100 mM EGTA, 140 mM NaCl). Lysates were diluted to 150 μg of DNA in 1.1 mL IP buffer (50 mM Tris pH 7.5, 10 mM EDTA, 140 mM NaCl, 1× protease inhibitors, 0.5% TX-100, 0.15% Deoxycholic acid) and pre-cleared for 1 h with 50 μL of Protein A Sepharose^®^ 4B (Invitrogen.) 250 μL of the pre-cleared lysates were dispensed to tubes containing the relevant antibodies (Millipore 07-352, Milipore 06-866 and Milipore 17-10046) or control antibody (rabbit serum; Millipore 17-10046) and incubated overnight, followed by addition of 40 μL of Protein A Sepharose and a further incubation for 1 h. The beads were washed twice with IP buffer, once with IP buffer plus 360 mM NaCl, once with LiCl buffer (0.25 M LiCl, 1 mM EDTA, 10 mM Tris pH 7.5, 0.5% TX-100) and once TE containing 0.2% TX-100, then resuspended in 100 μL TE with 0.2% TX-100 plus 1 μg of RNase A and incubated at 37 °C for 1 h, then overnight at 65 °C in the presence of 1% SDS followed by 2 h at 37 °C in the presence of Proteinase K. DNA was purified using the GenepHlow™ Gel/PCR kit and eluted into 100 μL of 10 mM Tris. 5 μL of this sample were analysed by qPCR with primers for the *ACT1*, *FLO1* or *FLO11* promoters using PowerUp SYBR Green 2× MasterMix (Applied Biosystems) and Applied Biosystems StepOne Plus™ thermocycler with StepOne Software. Three technical replicates were used to calculate a “fold over background” for each of the Histone H3, H3^AC^ and H4^AC^ immunoprecipitations, normalized to *ACT1* and then to signals from IPs with rabbit serum, using the formula 2^(CtIPserum−CtIP)^. The relative histone acetylation at each of these positions was determined by normalising the values from the H3, H3^AC^ and H4^AC^ immunoprecipitations to those from Histone 3. The results represent the average of 2 to 3 biological replicas for each strain/gene combination. Average values, standard deviations and *t* tests were calculated in Microsoft Excel^®^.

### Fluorescent microscopy

*W303* and isogenic *cac1∆*, *rrm3∆ cac1∆rrm3∆* strains were produced by replacement of *FLO11* ORF and promoter with a *GFP*-*KanMX* cassette as in [44]. All replacements were confirmed by PCR. Cell clusters of these strains were produced by serially diluting cells in 96-well trays and growing them without shaking for 24 h. Images were taken directly from the wells. Cells were also prepared by vigorous vortexing/pipetting of liquid cultures and spreading on slides. All images were taken with a Leica DM 6000B microscope with bright field or with the 469 nm filter. Images were processed and over-layered with Velocity™ software. The quantifying of GFP signals was done by subtracting the background pixel values (ROI with no cells) from the pixel values of identical ROI centred over isolated GFP+ or GFP− cells. For the calculation of the percent of GFP+ cells in Fig. 6b, only cells with 3 times higher signal as compared to the average signal in GFP− cells were counted.

### Analysis of FLO gene length variation

DNA from saturated liquid cultures was isolated and subjected to PCR with primers flanking *FLO1*, *FLO5*, *FLO9*, *FLO10* and *FLO11*. Primer sequences were exactly as in [6]. Primer sequences and coordinates in the genome are available upon request.

### Analysis of cell cycle

Exponentially growing cultures (OD_600_ = 1.0) were harvested, fixed in Ethanol and stained with propidium iodine as in [45]. Absorbance was measured by FC500 flow cytometer (Beckman–Coulter) and analysed was performed by FCS express 6 Plus software.

## Supplementary information

**Additional file 1: Table S1.** Strains used in this study. **Table S2.** Sedimentation scores in the presence of Nicotinamide (NAM). Sedimentation rates were determined by resting culture tubes and measuring the time needed for the clearance of the upper 50% of the culture (T^S50^). Values less than 1 indicate shorter T^S50^ relative to the non-treated sample. **Table S3.** List of PCR primers. **Figure S1.** All strains were simultaneously grown in YPD medium in a *Thermo-Scientific Multiskan* shaker-spectrophotometer. Time-course OD_600_ values are plotted. One of two independent experiments with all strains analysed in the same 96 well tray is shown. **Figure S2.** Exponentially growing cultures were harvested at OD_600_ = 1, stained with Propidium Iodine and analysed by flow cytometry. **Figure S3.** Genomic DNA was isolated from saturated liquid cultures and amplified by PCR with primers flanking the *FLO1*, *FLO5*, *FLO9* and *FLO11* genes. The PCR products were analysed on 1% agarose gels. **Figure S4.** Canavanine resistance in select strains. Four independent cultures of 10^7^ cells were spread on plates containing 60 μg/mL canavanine, the Can^R^ colonies were counted and plotted using “stock” graph by MS Excel^©^. The actual numbers of Can^R^ colonies on each plate are listed in the table below. The assay was performed only with the strains, which do not harbor the *can1-100* mutation. **Figure S5.** MMS sensitivity of the analysed strains. Exponentially growing cultures (OD_600_ = 1) of the strains shown on top were serially diluted and 5 microliter aliquots were spotted on YPD plates containing 0, 0.005, 0.01 and 0.02% MMS (shown on the right). One of two independent experiments is shown. **Figure S6.** Mating efficiency in double deletion mutants. Exponentially growing cultures (OD_600_ = 1) of the strains shown on the horizontal axis were serially diluted, mixed with 10^5^ *W303* cells of the opposing mating type in 0.25 mL of YPD medium and incubated for 4 h at 30 °C with gentle shaking. Five microliter aliquots were then spotted on SC dropout plates selecting for diploid cells and on plates selecting for both diploids and the tested haploids. SD dropout media were different for the different strains. The efficiency of mating was calculated as *per cent* of the number of diploids divided by the number of diploids/haploids. **Figure S7.** Sensitivity of chromatin to MNase digestion. 100 mL of exponentially growing cultures (OD_600_ = 1.6) of the strains shown on top of each panel were harvested and washed and cells were crushed by bead beating in Lysis buffer (140 mM NaCl, 50 mM Tris.HCl pH 7.6, 2 mM EDTA plus Protease Inhibitors). The extract was spun for 10 min at 13,000*g*, the chromatin pellet was resuspended in 1.5 mL MNase buffer plus Protease Inhibitors containing 6000 gels units of Microccocal nuclease (NEB) and incubated at 37 °C. Aliquots were removed at the times indicated and mixed with 1/10th volume STOP solution (10% SDS, 25 mM EDTA, 100 mM EGTA), DNA was purified and analysed on 1.2% agarose gels. The right-hand and the left-hand panel are from different experiments. At lease four experiments with each mutant strain in parallel with *W303* were performed.

## Data Availability

All data generated or analysed during this study are included in this published article and its Additional file.
