# Supplementary material for: Histone chaperones and the Rrm3p helicase regulate flocculation in S. cerevisiae
Source: Epigenetics Chromatin. 2019 Sep 23;12:56. doi: 10.1186/s13072-019-0303-8 (PMC6757361; doi:10.1186/s13072-019-0303-8)

ADDITIONAL FILE 1

Histone chaperones and the Rrm3p helicase regulate flocculation in *S. cerevisiae*

Hollie Rowlands, Kholoud Shaban, Barret Foster, Yannic Proteau, Krassimir Yankulov*
Department of Molecular and Cellular Biology, University of Guelph, Guelph, Canada
Correspondence: yankulov@uoguelph.ca

Table S1: Strains used in this study.

Strain	Genotype	Reference
BY4742	MATα his3Δ1 leu2Δ0 lys2Δ0 ura3Δ0	Open Biosystems
W303-1a	MATa leu2-3,112 trp1-1 can1-100 ura3-1 ade2-1 his3-11,15	NA
W303-1b	MATα leu2-3,112 trp1-1 can1-100 ura3-1 ade2-1 his3-11,15	NA
W303 flo11::GFP	W303 MATa flo11::GFP-KanMX	This study
asf1Δ	BY4742 asf1::KanMX	Open Biosystems
asf1Δ	ROY1246 MATα W303 asf1::HIS5+	[1]
asf1Δ flo11::GFP	W303 MATa asf1::HIS3 flo11::GFP-KanMX	This study
asf1Δcac1Δ	W303 MATa cac1::LEU2 asf1::KanMX6 hmr::GFP	[2]
asf1Δhda1Δ	MATa his3Δ1 leu2Δ0 ura3Δ0 asf1::HIS3 hda1::KanMX	This study
asf1Δhst1Δ	MATa his3Δ1 leu2Δ0 ura3Δ0 asf1::HIS3 hst1::KanMX	This study
asf1Δrrm3Δ	MATα his3Δ1 leu2Δ0 lys2Δ0 ura3Δ0 rrm3Δ::KanMX asf1Δ::HIS3	[3]
cac1Δ	BY4742 cac1::KanMX	Open Biosystems
cac1Δ	W303 cac1::KanMX	This study
cac1Δ	JLY030 MATα W303 cac1::LEU2	[4]
cac1Δ flo11::GFP	W303 MATa URA3-VIIL cac1::LEU2 flo11::GFP-KanMX	This study
cac1Δhda1Δ	MATα his3Δ1 leu2Δ0 ura3Δ0 cac1::LEU2 hda1::KanMX	This study
cac1Δrrm3Δ	MATa ade2-1 his3-11,15 leu2-3, 112 trp1-1 ura3-1 can1-100 cac1Δ::LEU2 rrm3Δ::TRP1	[3]
cac1Δrrm3Δ flo11::GFP	W303 MATa cac1::LEU2 rrm3::TRP1 flo11::GFP-KanMX	This study
cac1Δasf1Δ	PKY950 MATa W303 URA3- VIIL cac1::LEU2 asf1::HIS3	[5]
cac1Δhir1Δ	PKY969 MATa W303 cac1::hisG hir1::HIS3 URA3-VIIL	[6]
cac1Δsas2Δ	his3Δ1 leu2Δ0 lys2Δ0 ura3Δ0 cac1::LEU2 sas2::KanMX	[3]
cac1Δtof1Δ	MATα his3Δ1 leu2Δ0 lys2Δ0 ura3Δ0 cac1::LEU2 tof1::KanMX	[3]
English 1	wildtype brewing yeast	[7]
gcn5Δ	BY4742 gcn5::KanMX	Open Biosystems
hda1Δ	BY4742 hda1::KanMX	GE Dharmacon
hir1Δ	BY4742 hir1::KanMX	Open Biosystems
hir1Δ	PKY117 MATa W303 hir1::HIS3 URA3-VIIL	[8]
hir1Δhda1Δ	MATα his3Δ1 leu2Δ0 ura3Δ0 hir1::HIS3 hda1::KanMX	This study
hir1Δrrm3Δ	MATα his3Δ1 leu2Δ0 ura3Δ0 rrm3Δ::KanMX hir1Δ::HIS3	[3]
Hornindal 1	wild type brewing yeast	[9]
Hornindal 2	wild type brewing yeast	[9]

hst1Δ	*BY4742 hst1::KanMX*	GE Dharmacon
rpd3Δ	*MATa his3Δ1 leu2Δ0 lys2Δ0 ura3Δ0 rpd3::KanMX*	This study
rrm3Δ	*rrm3::HIS3 URA3-VIIL-tel*	[10]
rrm3Δ	*rrm3::TRP1 YPH499 (MATa, ura3-52, lys2-801, ade2-101, rpl-Δ63,his3Δ200, leu2Δ1*	[10]
rrm3Δ	*BY4742 rrm3::KanMX*	Open Biosystems
rrm3Δ flo11::GFP	*rrm3::TRP1 flo11::GFP-KanMX*	This study
rrm3Δhda1Δ	*MATα his3Δ1 leu2Δ0 ura3Δ0 rrm3::HIS3 hda1::KanMX*	This study
rrm3Δtof1Δ	*MATα his3Δ1 leu2Δ0 ura3Δ0 rrm3::HIS3 tof1::KanMX*	[3]
rtt106Δ	*BY4742 rtt106::KanMX*	Open Biosystems
sas2Δ	*BY4742 sas2::KanMX*	Open Biosystems
sir2Δ	*LPY11 MATa sir2::HIS3 leu2-3,112 trp1-1 can1-100 ura3-1 ade2-1 his3-11,15*	[11]
tof1Δ	*BY4742 tof1::KanMX*	Open Biosystems
POL30	*pol30Δ pBL230-POL30 ade2-1 ura3-1 his3-11,15 trp1-1 leu2-3,112 can1-100 MATα*	[12]
pol30-6	*pol30Δ pBL230-pol30-6 ade2-1 ura3-1 his3-11,15 trp1-1 leu2-3,112 can1-100 MATα*	[12]
pol30-79	*pol30Δ pBL230-pol30-79 ade2-1 ura3-1 his3-11,15 trp1-1 leu2-3,112 can1-100 MATα*	[12]
EM93	*feral strain*	C. Boone collection, University of Toronto

Table S2: Sedimentation scores in the presence of Nicotinamide (NAM). Sedimentation rates were determined by resting culture tubes and measuring the time needed for the clearance of the upper 50% of the culture (T^{S50}). Values less than 1 indicate shorter T^{S50} relative to the non-treated sample.

		average T^{S50}		STD		T^{S50}: time time needed for the clearance of the upper 50% of the culture						
strain		2 mM NAM	5 mM NAM	2 mM NAM	5 mM NAM	2 mM NAM			5 mM NAM			0 mM NAM
BY4742	*BY4742*	1	1	0	0	1	1	1	1	1	1	1
W303	*W303*	1	1	0	0	1	1	1	1	1	1	1
asf1Δ	*asf1Δ BY4742*	0.4333	0.353	0.058	0.04	0.4	0.5	0.4	0.4	0.33	0.33	1
asf1Δ	*asf1Δ W303*	0.475	0.267	0.318	0.029	0.7	0.25		0.3	0.25	0.25	1
cac1Δ	*cac1Δ BY4742*	0.4333	0.327	0.058	0.075	0.4	0.5	0.4	0.25	0.33	0.4	1
cac1Δ	*cac1Δ W303*	0.4333	0.303	0.058	0.046	0.4	0.5	0.4	0.33	0.25	0.33	1
hir1Δ	*hir1Δ BY4742*	0.75	0.45	0.071	0.071	0.8	0.7		0.5	0.4		1
hir1Δ	*hir1Δ W303*	0.7333	0.333	0.058	0.058	0.7	0.8	0.7	0.4	0.3	0.3	1
rtt106Δ	*rtt106Δ BY4742*	0.65	0.315	0.071	0.021	0.7	0.6		0.3	0.33		1
rrm3Δ	*rrm3Δ BY4742*	0.8	0.767	0.1	0.231	0.7	0.8	0.9	0.5	0.9	0.9	1
rrm3Δ	*rrm3Δ W303*	1	0.75	0	0.071	1	1		0.7	0.8		1
hst1Δ	*hst1Δ BY4742*	1	1	0	0	1	1		1	1		1
tof1Δ	*tof1Δ BY4742*	1	1	0	0	1	1		1	1		1
sir2Δ	*sir2Δ BY4742*	1	1	0	0	1	1		1	1		1
gcn5Δ	*gcn5Δ BY4742*	1	1	0	0	1	1		1	1		1

Table S3: List of PCR primers.

Primer Set	F sequence	R sequence	Coordinates	Application	Reference
FLO1 promoter	TGTGGAACCTTCTACAGTACTTCGG	TTTGAGTGCCTTTCAACAATTTCAGACTT	chrI:202955-203050	ChIP	[13]
FLO11 promoter	GGGATTCAAGGCATCATCGC	TCGAACACGGACATTCCTCA	chrIX:394141-394050	ChIP	This study
ACT1	CTCCACCACTGCTGAAAGAGAA	CCAAGGCGACGTAACATAGTTTT	chrVI:53321-53279	ChIP, RT-qPCR	[14]
FLO1	ACTGTCACTGGAACCAATGGC	CCTGAAGATGATGATGACAAACTGG	chrI:206541-206617	RT-qPCR	This study
FLO5	GTGAGGGTTTGATTACTACAACTACCG	TGCTGATTAGACCTTCACTGG	chrVIII:526746-526878; 526611-526637; 526476-526878	RT-qPCR	This study
FLO1/FLO9	TCCAACCAGTGAAGGTTTGGTT	AGCTGGTGATTTGTCCTGAAGA	chrI:206946-207118; chrI:25981-25812	RT-qPCR	This study
FLO10	TGACCGGCCTATTTTTGCTATC	CTGCATCAGCATAACCATAGGCCAT	chrXI:644393-644542	RT-qPCR	This study
FLO11	GGCACATGGACCAGCAAAATAT	GTGTGGCATAAACTTCAAAAGTGGC	chrIX:393611-393381	RT-qPCR	This study
FLO1 ORF	ATCGCTATATGTTTTTGGCAGTCTTTA	TTAAATAATTGCCAGCAATAAGGACG	chrI:203416-208016	length variation	[15]
FLO5 ORF	GCACACCACTGCATATTTTTGGTAA	TTAAATAATTGCCAGCAATAAGGACG	chrVIII:525401-528594	length variation	[15]
FLO9 ORF	TTATTGTTTACTACTAGCCATCGTCACA	TTAAATAATTGCCAGCAATAAGGACG	chrI:27954-24025	length variation	[15]
FLO10 ORF	CTGAATATAGCGCTTCCCAGGTT	GGCAATGAAAATACTAATACCACTATTGGTT	chrXI:646660-649811	length variation	[15]
FLO11 ORF	CACTTTTGAAGTTTATGCCACACAAG	TTAGAATACAACTGGAAGAGCGAGTAGC	chrIX:393292-389599	length variation	[15]

REFERENCES TO TABLES S1 AND TABLE S3

1. Tyler JK, Adams CR, Chen SR, Kobayashi R, Kamakaka RT, Kadonaga JT. The RCAF complex mediates chromatin assembly during DNA replication and repair. Nature. 1999;402(6761):555-60. doi: 10.1038/990147. PubMed PMID: 10591219.
2. Huang S, Zhou H, Katzmann D, Hochstrasser M, Atanasova E, Zhang Z. Rtt106p is a histone chaperone involved in heterochromatin-mediated silencing. Proc Natl Acad Sci U S A. 2005;102(38):13410-5. Epub 2005/09/15. doi: 0506176102 [pii]

10.1073/pnas.0506176102. PubMed PMID: 16157874; PubMed Central PMCID: PMC1224646.
3. Wyse B, Oshidari R, Rowlands H, Abbasi S, Yankulov K. RRM3 regulates epigenetic conversions in Saccharomyces cerevisiae in conjunction with Chromatin Assembly Factor I. Nucleus. 2016;7(4):405-14. doi: 10.1080/19491034.2016.1212796. PubMed PMID: 27645054.
4. Linger J, Tyler JK. The yeast histone chaperone chromatin assembly factor 1 protects against double-strand DNA-damaging agents. Genetics. 2005;171(4):1513-22. Epub 2005/09/07. doi: genetics.105.043000 [pii]

10.1534/genetics.105.043000. PubMed PMID: 16143623; PubMed Central PMCID: PMC1456080.
5. Krawitz DC, Kama T, Kaufman PD. Chromatin assembly factor I mutants defective for PCNA binding require Asf1/Hir proteins for silencing. Mol Cell Biol. 2002;22(2):614-25. Epub 2002/01/05. PubMed PMID: 11756556; PubMed Central PMCID: PMC139734.
6. Sharp JA, Franco AA, Osley MA, Kaufman PD. Chromatin assembly factor I and Hir proteins contribute to building functional kinetochores in S. cerevisiae. Genes Dev. 2002;16(1):85-100. doi: 10.1101/gad.925302. PubMed PMID: 11782447; PubMed Central PMCID: PMC155315.
7. Gallone B, Steensels J, Prahl T, Soriaga L, Saels V, Herrera-Malaver B, et al. Domestication and Divergence of Saccharomyces cerevisiae Beer Yeasts. Cell. 2016;166(6):1397-410 e16. doi: 10.1016/j.cell.2016.08.020. PubMed PMID: 27610566; PubMed Central PMCID: PMCPMC5018251.
8. Kaufman PD, Cohen JL, Osley MA. Hir proteins are required for position-dependent gene silencing in Saccharomyces cerevisiae in the absence of chromatin assembly factor I. Mol Cell Biol. 1998;18(8):4793-806. PubMed PMID: 9671489; PubMed Central PMCID: PMC109065.
9. Preiss R, Tyrawa C, Krogerus K, Garshol LM, van der Merwe G. Traditional Norwegian Kveik Are a Genetically Distinct Group of Domesticated Saccharomyces cerevisiae Brewing Yeasts. Front Microbiol. 2018;9:2137. doi: 10.3389/fmicb.2018.02137. PubMed PMID: 30258422; PubMed Central PMCID: PMCPMC6145013.
10. Ivessa AS, Zhou JQ, Schulz VP, Monson EK, Zakian VA. Saccharomyces Rrm3p, a 5' to 3' DNA helicase that promotes replication fork progression through telomeric and subtelomeric DNA. Genes Dev. 2002;16(11):1383-96. Epub 2002/06/07. doi: 10.1101/gad.982902. PubMed PMID: 12050116; PubMed Central PMCID: PMC186315.
11. Koch MR, Pillus L. The glucanosyltransferase Gas1 functions in transcriptional silencing. Proc Natl Acad Sci U S A. 2009;106(27):11224-9. doi: 10.1073/pnas.0900809106. PubMed PMID: 19541632; PubMed Central PMCID: PMCPMC2708717.
12. Zhang Z, Shibahara K, Stillman B. PCNA connects DNA replication to epigenetic inheritance in yeast. Nature. 2000;408(6809):221-5. Epub 2000/11/23. doi: 10.1038/35041601. PubMed PMID: 11089978.
13. Church M, Smith KC, Alhussain MM, Pennings S, Fleming AB. Sas3 and Ada2(Gcn5)-dependent histone H3 acetylation is required for transcription elongation at the de-repressed FLO1 gene. Nucleic acids research. 2017;45(8):4413-30. PubMed PMID: Medline:28115623.
14. Smukalla S, Caldara M, Pochet N, Beauvais A, Guadagnini S, Yan C, et al. FLO1 is a variable green beard gene that drives biofilm-like cooperation in budding yeast. Cell. 2008;135(4):726-37. doi: 10.1016/j.cell.2008.09.037. PubMed PMID: 19013280; PubMed Central PMCID: PMCPMC2703716.
15. Verstrepen KJ, Jansen A, Lewitter F, Fink GR. Intragenic tandem repeats generate functional variability. Nat Genet. 2005;37(9):986-90. doi: 10.1038/ng1618. PubMed PMID: 16086015; PubMed Central PMCID: PMCPMC1462868.

SUPPLEMENTAL FIGURE LEGENDS

Figure S1: All strains were simultaneously grown in YPD medium in a *Thermo-Scientific Multiskan* shaker-spectrophotometer. Time-course OD_{600} values are plotted. One of two independent experiments with all strains analysed in the same 96 well tray is shown.

Figure S2: Exponentially growing cultures were harvested at OD_{600}=1, stained with Propidium Iodine and analysed by flow cytometry.

Figure S3. Genomic DNA was isolated from saturated liquid cultures and amplified by PCR with primers flanking the *FLO1, FLO5, FLO9* and *FLO11* genes. The PCR products were analysed on 1% agarose gels.

Figure S4. Canavanine resistance in select strains. Four independent cultures of 10^7 cells were spread on plates containing 60 µg/ml canavanine, the Can^R colonies were counted and plotted using "stock" graph by MS Excel©. The actual numbers of Can^R colonies on each plate are listed in the table below. The assay was performed only with the strains, which do not harbor the *can1-100* mutation.

Figure S5. MMS sensitivity of the analysed strains. Exponentially growing cultures (OD_{600}=1) of the strains shown on top were serially diluted and 5 microliter aliquots were spotted on YPD plates containing 0, 0.005, 0.01 and 0.02 % MMS (shown on the right). One of two independent experiments is shown.

Figure S6. Mating efficiency in double deletion mutants. Exponentially growing cultures (OD_{600}=1) of the strains shown on the horizontal axis were serially diluted, mixed with 10^5 *W303* cells of the opposing mating type in 0.25 ml of YPD medium and incubated for 4 hours at 30°C with gentle shaking. Five microliter aliquots were then spotted on SC dropout plates selecting for diploid cells and on plates selecting for both diploids and the tested haploids. SD dropout media were different for the different strains. The efficiency of mating was calculated as *per cent* of the number of diploids divided by the number of diploids/haploids.

Figure S7. Sensitivity of chromatin to MNase digestion. 100 ml of exponentially growing cultures (OD_{600}=1.6) of the strains shown on top of each panel were harvested and washed and cells were crushed by bead beating in Lysis buffer (140 mM NaCl, 50 mM Tris.HCl pH 7.6, 2 mM EDTA plus Protease Inhibitors). The extract was spun for 10 min at 13000g, the chromatin pellet was resuspended in 1.5 ml MNase buffer plus Protease Inhibitors containing 6000 gels units of Micrococcal nuclease (NEB) and incubated at 37°C. Aliquots were removed at the times indicated and mixed with $1/10^{th}$ volume STOP solution (10%SDS, 25 mM EDTA, 100 mM EGTA), DNA was purified and analysed on 1.2% agarose gels. The right-hand and the left-hand panel are from different experiments. At lease four experiments with each mutant strain in parallel with *W303* were performed.

Figure S1

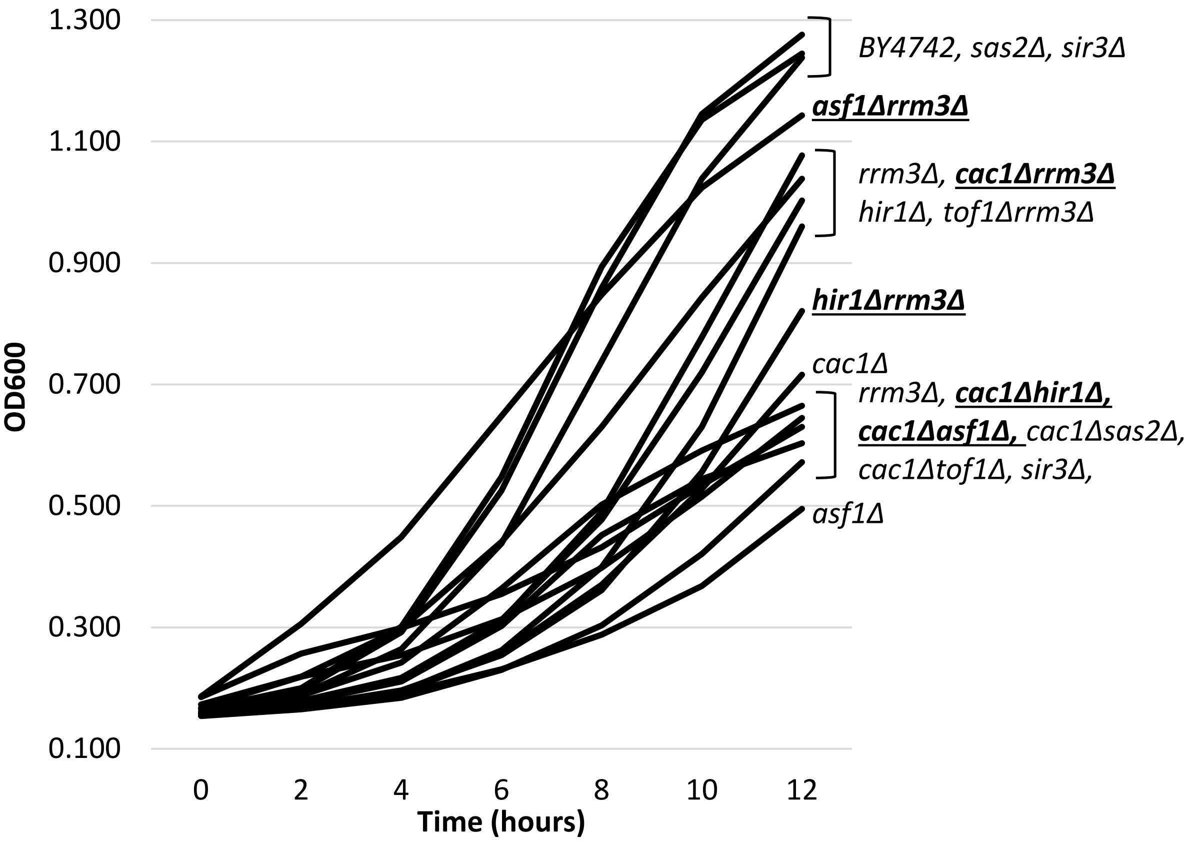
1.300
1.100
0.900
0.700
0.500
0.300
0.100
OD600
0
2
4
6
8
10
12
Time (hours)
BY4742, sas2Δ, sir3Δ
asf1Δrrm3Δ
rrm3Δ, cac1Δrrm3Δ
hir1Δ, tof1Δrrm3Δ
hir1Δrrm3Δ
cac1Δ
rrm3Δ, cac1Δhir1Δ,
cac1Δasf1Δ, cac1Δsas2Δ,
cac1Δtof1Δ, sir3Δ,
asf1Δ

Figure S2

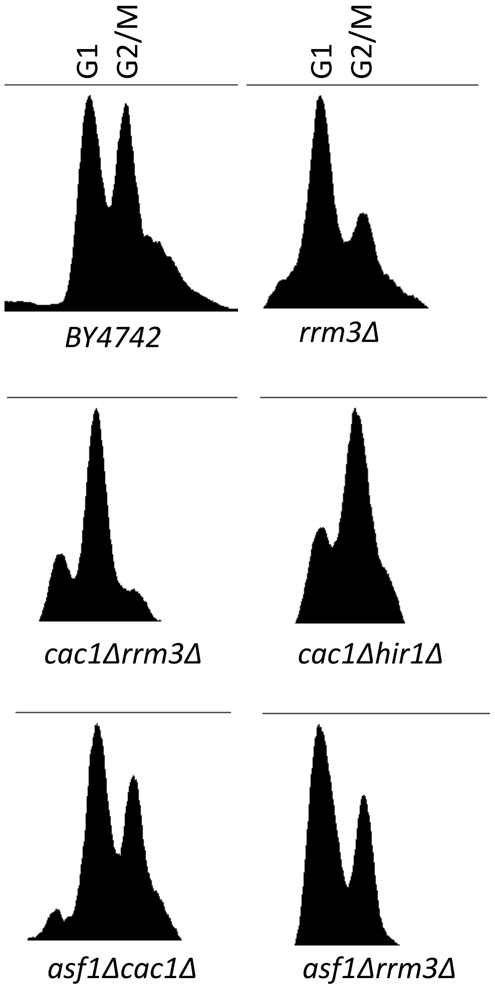

Figure S3

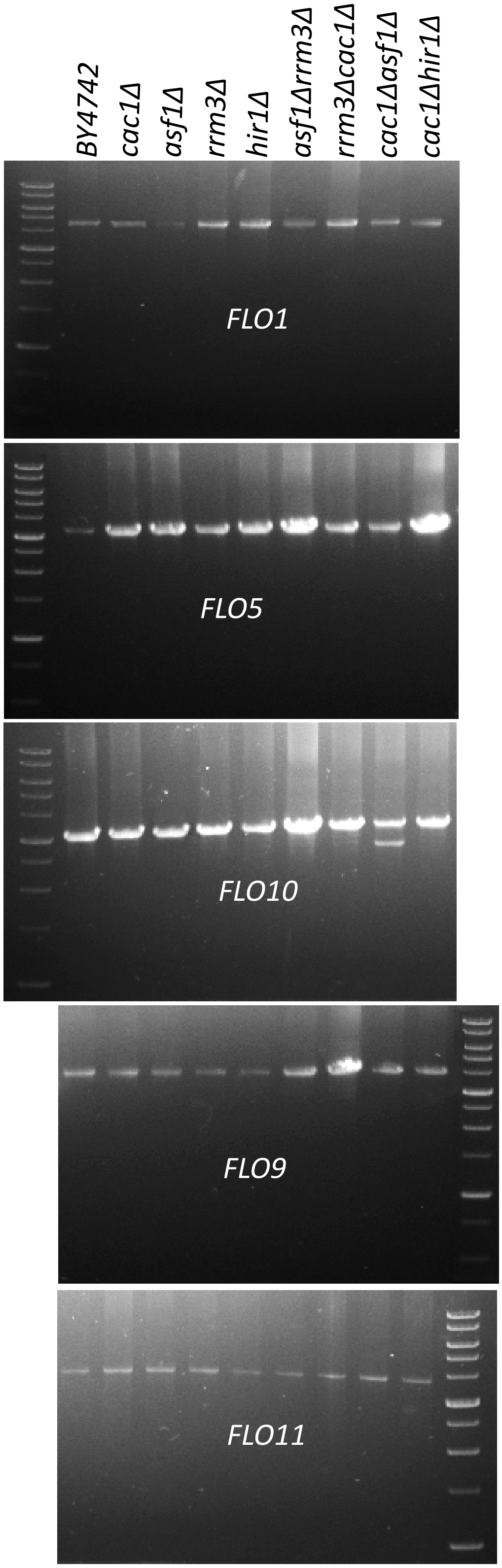

Figure S4

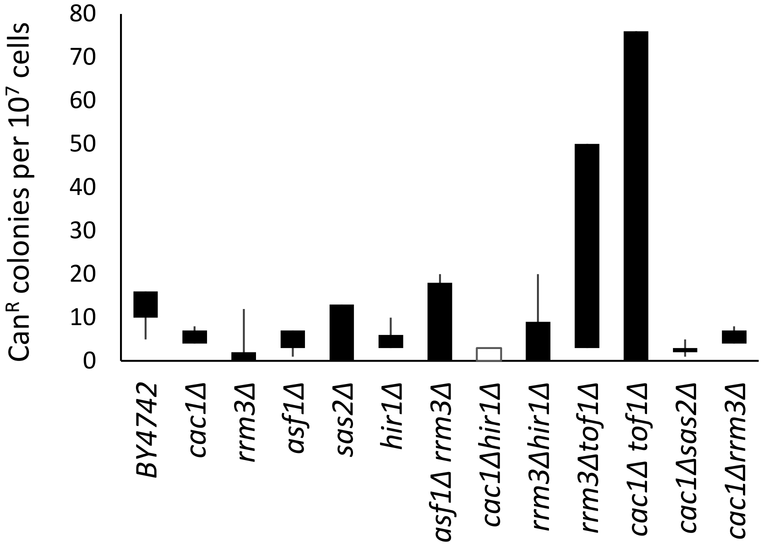

Strain	plate 1	plate 2	plate 3	plate 4
BY4742	16	5	6	10
cac1Δ	7	7	8	4
rrm3Δ	2	11	12	0
asf1Δ	7	1	2	3
sas2Δ	13	8	9	0
hir1Δ	6	4	10	3
asf1Δ rrm3Δ	18	16	20	0
cac1Δhir1Δ	0	1	0	3
rrm3Δhir1Δ	9	20	18	0
rrm3Δtof1Δ	50	42	30	3
cac1Δ tof1Δ	76	8	35	0
cac1Δsas2Δ	3	1	5	2
cac1Δrrm3Δ	7	7	8	4

Figure S5

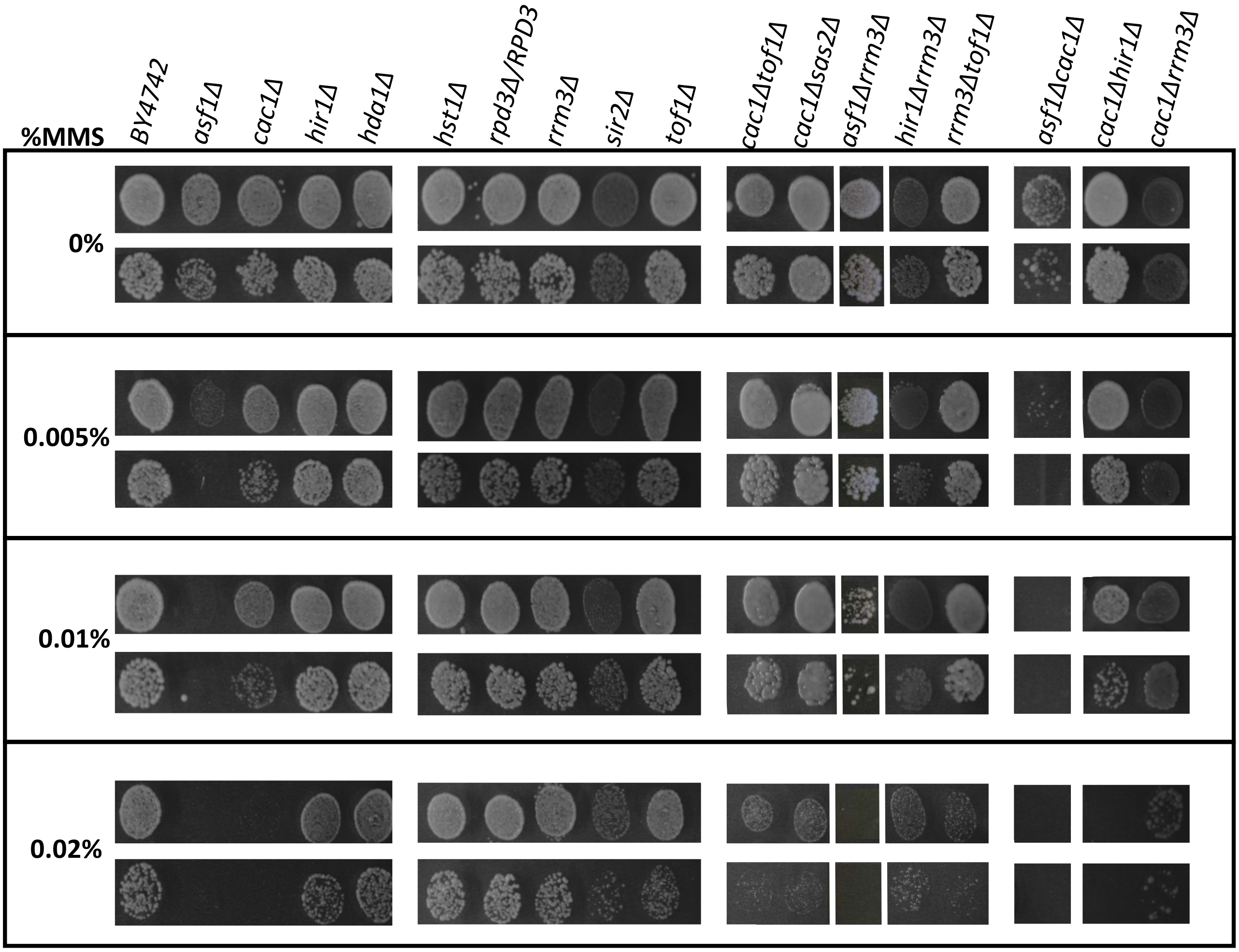
%MMS
BY4742
asf1Δ
cac1Δ
hir1Δ
hda1Δ
hst1Δ
rpd3Δ/RPD3
rrm3Δ
sir2Δ
tof1Δ
cac1Δtof1Δ
cac1Δsas2Δ
asf1Δrrm3Δ
hir1Δrrm3Δ
rrm3Δtof1Δ
asf1Δcac1Δ
cac1Δhir1Δ
cac1Δrrm3Δ
0%
0.005%
0.01%
0.02%

Figure S6

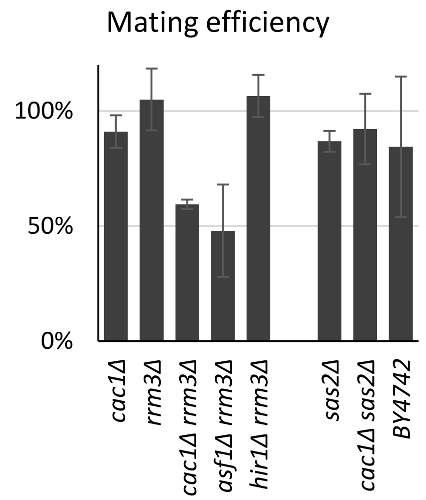

Figure S7

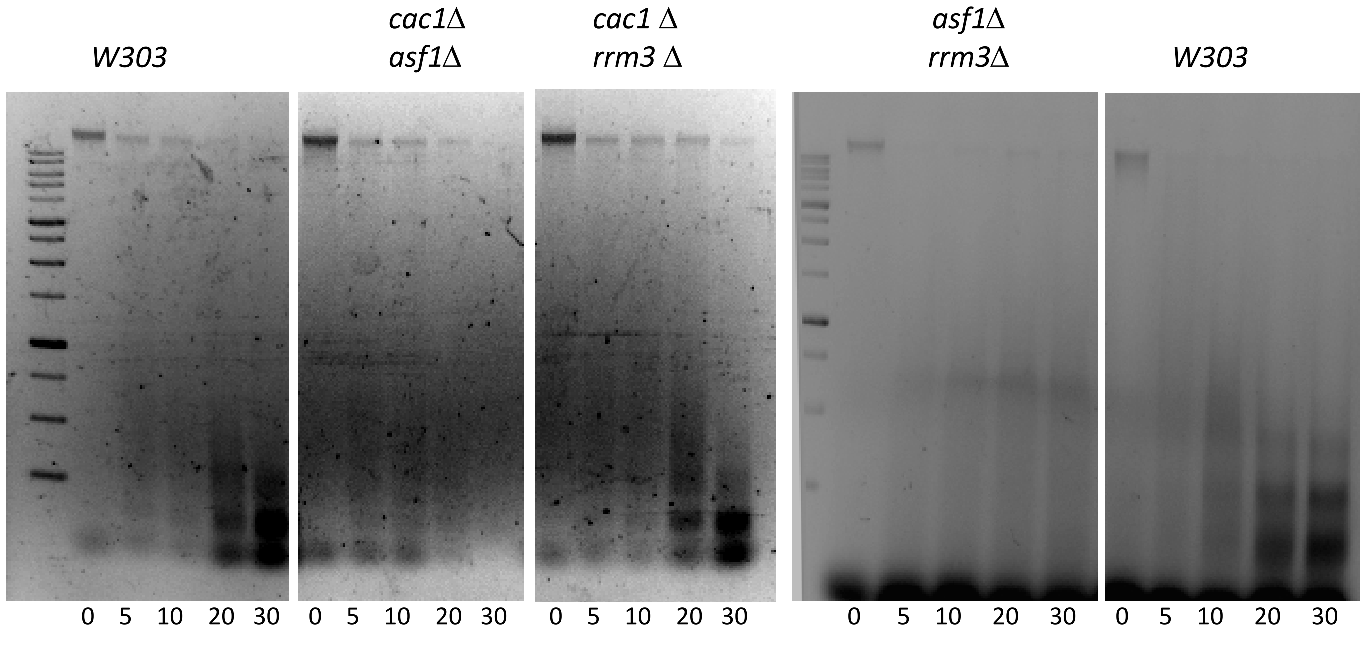

Supplement: Supplementary file 1 — Additional file 1: Table S1. Strains used in this study. Table S2. Sedimentation scores in the presence of Nicotinamide (NAM). Sedimentation rates were determined by resting culture tubes and measuring the time needed for the clearance of the upper 50% of the culture (TS50). Values less than 1 indicate shorter TS50 relative to the non-treated sample. Table S3. List of PCR primers. Figure S1. All strains were simultaneously grown in YPD medium in a Thermo-Scientific Multiskan shaker-spectrophotometer. Time-course OD600 values are plotted. One of two independent experiments with all strains analysed in the same 96 well tray is shown. Figure S2. Exponentially growing cultures were harvested at OD600 = 1, stained with Propidium Iodine and analysed by flow cytometry. Figure S3. Genomic DNA was isolated from saturated liquid cultures and amplified by PCR with primers flanking the FLO1, FLO5, FLO9 and FLO11 genes. The PCR products were analysed on 1% agarose gels. Figure S4. Canavanine resistance in select strains. Four independent cultures of 107 cells were spread on plates containing 60 μg/mL canavanine, the CanR colonies were counted and plotted using “stock” graph by MS Excel©. The actual numbers of CanR colonies on each plate are listed in the table below. The assay was performed only with the strains, which do not harbor the can1-100 mutation. Figure S5. MMS sensitivity of the analysed strains. Exponentially growing cultures (OD600 = 1) of the strains shown on top were serially diluted and 5 microliter aliquots were spotted on YPD plates containing 0, 0.005, 0.01 and 0.02% MMS (shown on the right). One of two independent experiments is shown. Figure S6. Mating efficiency in double deletion mutants. Exponentially growing cultures (OD600 = 1) of the strains shown on the horizontal axis were serially diluted, mixed with 105 W303 cells of the opposing mating type in 0.25 mL of YPD medium and incubated for 4 h at 30 °C with gentle shaking. Five microliter aliquots were th [file 13072_2019_303_MOESM1_ESM.pdf]
